# Supplementary material for: LIDER: cell embedding based deep neural network classifier for supervised cell type identification
Source: PeerJ. 2023 Aug 16;11:e15862. doi: 10.7717/peerj.15862 (PMC10439717; doi:10.7717/peerj.15862)
Supplement: Supplemental Information 1 [file peerj-11-15862-s001.pdf]

Supplemental Data to

LIDER: cell embedding based deep neural network classifier for supervised cell type identification

Yachen Tang, Xuefeng Li, Mingguang Shi*

We developed a multi-class scRNA-seq classifier with stacked denoising autoencoder for cell type identification.

(a) step1: ScRNA-seq data are collected and z-score transformed.

(b)step2: LIDER generates cell embeddings using stacked denoising autoencoder. Stacked denoising autoencoders (SDAE) are developed by multiple stacking layers of denoising autoencoders. In this analysis, the selected features of 1000 dimension from single-cell data as low-dimensional representations were derived to develop a classifier for identifying cell types.

(c) step3: A deep neural network classifier is then developed by using Adam algorithm for classification tasks. Adam is a promising stochastic optimization algorithm for first-order gradient-based optimization with stochastic objective functions. Let $\boldsymbol{f(\theta)}$ be a stochastic objective function with respect to parameters $\boldsymbol{\theta} = (\boldsymbol{W}, \boldsymbol{b})$, our aim is to minimize the expected value of this objective function. In this analysis, the cross-entropy function (1) is the objective functions.

$$\boldsymbol{L = -\sum_{i=1}^{n} \sum_{j=1}^{J} y_{ji}\, log \bar{y}_{ji}} \tag{1}$$

Gradients are derived with stochastic objective at timestep t according to the following equation:

$$\boldsymbol{g_t = \nabla_\theta f_t(\theta_{t-1})} \tag{2}$$

where parameters $\boldsymbol{\theta_{t-1}} = (\boldsymbol{W_{t-1}}, \boldsymbol{b_{t-1}})$.
Biased first moment estimate at timestep *t* updates as follows:

$$\boldsymbol{m_t = \beta_1 m_{t-1} + (1 - \beta_1) g_t} \tag{3}$$

where $\boldsymbol{\beta_1} \in [\boldsymbol{0}, \boldsymbol{1}]$ is an exponential decay rate for the moment estimate, $\boldsymbol{m_{t-1}}$represents biased first moment estimate at timestep $(\boldsymbol{t-1})$ **and** $\mathrm{m}_0 \leftarrow 0$ respectively. Biased second raw moment estimate updates at timestep *t* as

follows:

$$\boldsymbol{V_t = \beta_2 V_{t-1} + (1-\beta_2) g_t^2} \tag{4}$$

where $\boldsymbol{\beta_2} \in [\mathbf{0}, \mathbf{1}]$ is an exponential decay rate for the moment estimate, $\boldsymbol{v_{t-1}}$represents biased second raw moment estimate at timestep $(\boldsymbol{t-1})$ **and** $v_0 \leftarrow 0$ respectively. Bias-corrected first moment estimate is computed as follows:

$$\boldsymbol{\widehat{m_t} = m_t/(1-\beta_1^t)} \tag{5}$$

Bias-corrected second raw moment estimate is computed as follows:

$$\boldsymbol{\widehat{V}_t = V_t/(1-\beta_2^t)} \tag{6}$$

Parameters $\boldsymbol{\theta}$ update as follows:

$$\boldsymbol{\theta_t = \theta_{t-1} - \alpha * \widehat{m}_t/(\sqrt{\widehat{V}_t} + \varepsilon)} \tag{7}$$

where $\boldsymbol{\alpha}$ is a step size and $\boldsymbol{\varepsilon}$ is 10^{-8} respectively.
